# The Effect of Breastfeeding on Intelligence Quotient and Social Intelligence Among Seven- to Nine-Year-Old Girls: A Pilot Study

**DOI:** 10.3389/fnut.2022.726042

**Published:** 2022-02-18

**Authors:** Danyah Abdullah AlThuneyyan, Faten Fahad AlGhamdi, Ragad Nasser AlZain, Zainab Sami AlDhawyan, Haya Fahad Alhmly, Tunny Sebastian Purayidathil, Yasmin Yussuf AlGindan, Ahmed Amr Abdullah

**Affiliations:** ^1^Department of Clinical Nutrition, Imam Abdulrahman Bin Faisal University, Dammam, Saudi Arabia; ^2^The University Counseling Center at Imam Abdulrahman Bin Faisal University, Dammam, Saudi Arabia

**Keywords:** breastfeeding, bottle-feeding, social intelligence, intelligence quotient (IQ), body mass index, Saudi Arabia

## Abstract

**Background:**

Breastfeeding is an optimal infant feeding method that provides adequate nutrients, achieves healthy growth and development, and enhances the health status of both infants and mothers. Breast milk contains a variety of substances that might positively affect cognition and the development of children's psychomotor abilities.

**Objective:**

This study aimed to examine the variations in intelligence quotient (IQ), social intelligence (SI), and body mass index (BMI) among 7- to 9-year-old girls who were exclusively breastfed, exclusively bottle-fed, or mixed-fed during their first 6 months of life.

**Methods:**

This study involved 111 healthy girls, aged 7 to 9 years, who were recruited from nine government and private schools in Dammam, Kingdom of Saudi Arabia. Raven's Coloured Progressive Matrices were used to measure the participants' IQs, and the Vineland Social Maturity Assessment was used to measure their SI through individual interviews. Anthropometric measurements were obtained using standard methods.

**Results:**

The breastfed group showed a greater number of above-average IQ test scores (35 vs. 23%; *P* = 0.479) and better SI scores (78 vs. 55%; *P* = 0.066) compared with the bottle-fed group. The number of girls with normal BMIs was significantly higher in the breastfed group than in the bottle-fed (68 vs. 41%; *P* = 0.045) or mixed-fed groups.

**Conclusion:**

Exclusively breastfed girls had higher IQ and SI results compared with bottle-fed girls. However, unlike the BMI differences, these results were not statistically significant. This study provides fundamental observational data and can be further modified for use on a larger national-scale level.

## Background

The Academy of Nutrition and Dietetics suggests that exclusive breastfeeding provides ideal nutrition and health defence for the first 6 months of life, and breastfeeding with complementary food from 6 months until at least 12 months of age is considered the optimal nutrition for infants (1). Worldwide, only 41% of infants younger than 6 months of age are exclusively breastfed. The rates of fully breastfed newborns are ~52% in South Asia, 22% in East Asia, 33% in the Middle East, 32% in Eastern Europe and Central Asia, and 26% in North America (www.Unicef.org) (2). In Saudi Arabia, 17 cross-sectional studies were identified and reviewed. The results showed that the most common feeding method was mixed feeding (57.9–88.6%), whereas exclusive breastfeeding rates in the studies ranged from 0.8 to 43.9% (3).

The benefits of breastfeeding appear supreme in studies of young children. It contains a variety of anti-inflammatory, immunomodulatory, and antimicrobial substances that positively affect cognition and psychomotor development in children (4).

It is now evident that the gut microbiome plays a critical role in infant development, and the mode of feeding can alter the microbial communities of the gastrointestinal tract (5). Formula milk, given in small amounts during breastfeeding, can change the structure and relative abundance of the bacterial communities normally found in the gut of a breastfed infant. The introduction of formula disturbs the colonisation and proliferation of neonatal intestinal microbiota and may decrease the benefits of exclusive human-milk feeding (6).

Several studies have reported that breastfed children experience health benefits, such as a lower prevalence of overweight status or obesity, lower blood pressure, and lower total cholesterol, as well as higher levels of school achievement and a high degree of intelligence. However, others have failed to support such associations (7). An Australian longitudinal cohort study examined the effect of predominant breastfeeding for <6 vs. ≥6 months on motor proficiency at 10, 14, and 17 years. Results showed that breastfeeding for ≥6 months improved optimal neuromotor outcomes (8). Other studies have investigated the impact of breastfeeding on body mass index (BMI). A systemic review and meta-analysis published in 2012 assessed the risk factors for childhood overweight status during infancy. It included 10 prospective studies and determined that breastfeeding at any time in the child's first year reduced the adjusted odds of being overweight in childhood by 15% compared with non-breastfed children (9). Most studies in this field have focused on explaining the association between breastfeeding and cognitive and psychosocial abilities. However, few studies have examined the three outcomes together in a single study, and to our knowledge, none have been conducted in Saudi Arabia. This study investigated the differences in intelligence quotient (IQ), social intelligence (SI), and BMI among Saudi girls from Dammam, Saudi Arabia, aged 7 to 9 years, who were exclusively breastfed, mixed-fed, or exclusively bottle-fed.

## Methods

### Study Design

This cross-sectional study was performed from December 2018 to September 2019 in the city of Dammam, Saudi Arabia. Five governmental schools and four private schools took part in the study, which covered different social classes. Participants who were non-Saudi, had hearing and sight problems, had mental difficulties, or lived away from their mothers were excluded. Initially, this study included 127 students chosen randomly from nine different schools. Multistage probability sampling was employed to select the sample from each area of the city.

**Figure 1:**
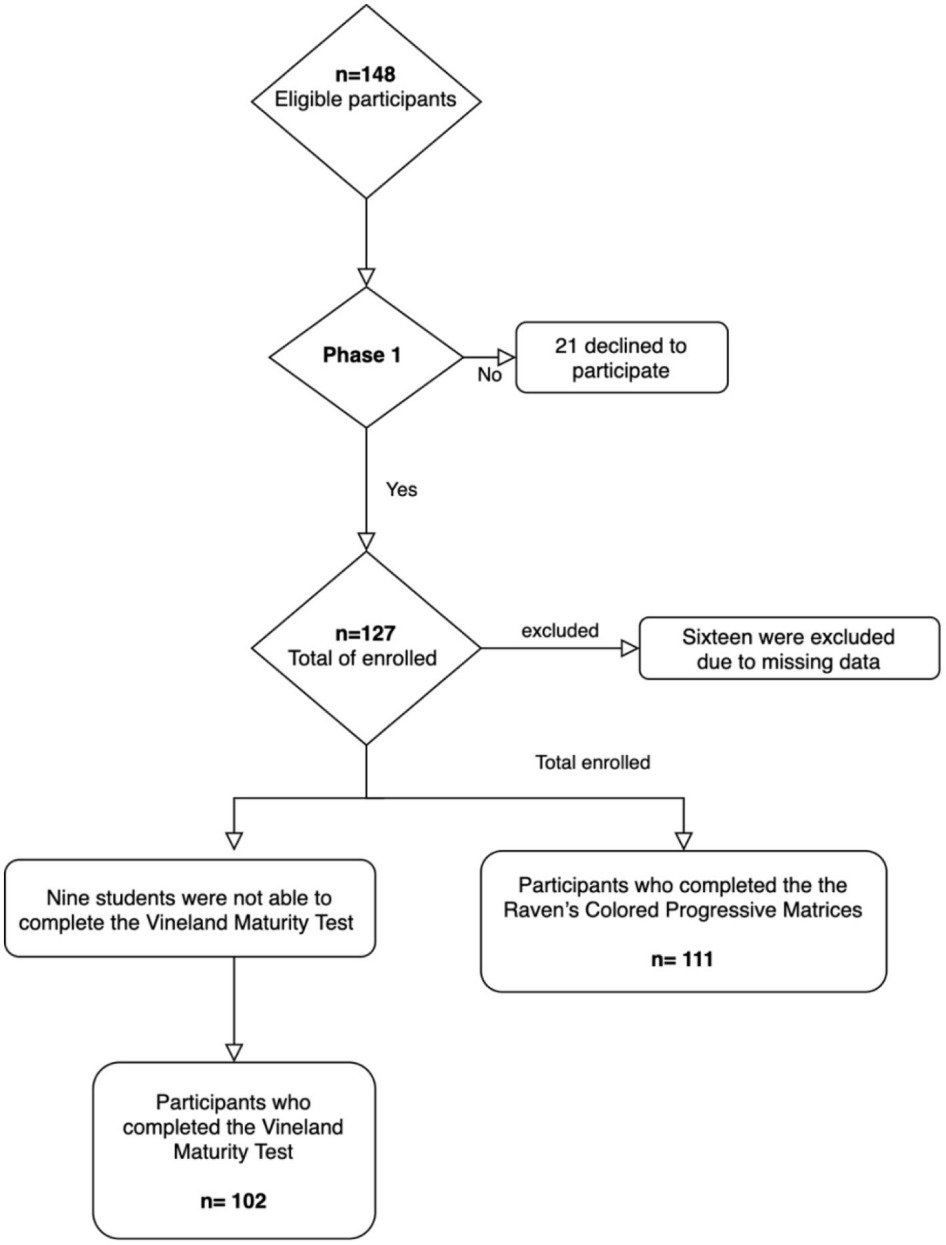
Flowchart of participant selection.

Sixteen students had missing data or were unable to complete the tests, and so were excluded. Overall, 111 participants were included in the data analysis for IQ and BMI, and 102 students were included in the SI data analysis (Figure 1). Approval from the Department of Clinical Nutrition and Institutional Review Board of Imam Abdulrahman Bin Faisal University and the Ministry of Education was obtained (IRB:−2018-03-244) before conducting the study. The parents also signed the consent forms.

### Data Collection

Gestational age was categorised into very premature ( ≤ 7 months), premature ( ≤ 8 months), and term newborn (≥9 months). The type of feeding was defined according to the WHO as exclusively breastfeeding for the first 6 months of life, exclusively bottle-feeding, or mixed-feeding (10). Socioeconomic status (SES) was classified according to the Saudi Arabian household expenditures and incomes (low, <5,000 Saudi Riyals [SAR]; moderate, 5,000–10,000 SAR; high, >10,000 SAR) (https://www.stats.gov.sa/) (11). Data regarding parent education level (no education, elementary, intermediate, high school, college, or postgraduate) were collected by the researchers and are presented in Table 1.

**Table 1:** Demographic data of the study participants.

	**Total**		**Breastfeeding** **(*****n*** **=** **37)**		**Bottle feeding** **(*****n*** **=** **22)**		**Mixed** **feeding** **(*****n*** **=** **52)**	
	***N***	**%**	***N***	**%**	***N***	**%**	***N***	**%**
**Age in years**								
7	3	3	1	3	0	0	2	4
8	88	79	30	81	20	91	38	73
9	20	18	6	16	2	9	12	23
**Economic status**								
Low	18	16	6	16	5	23	7	14
Moderate	45	41	17	46	7	32	21	40
High	48	43	14	38	10	45	24	46
**Mothers' education level**								
No education	2	2	0	0	1	5	1	2
Elementary	11	10	4	11	1	5	6	11
Intermediate	10	9	3	8	3	13	4	8
High school	34	31	14	38	4	18	16	31
College	48	43	15	40	11	50	22	42
Higher education	6	5	1	3	2	9	3	6
**School**								
Private	49	44	12	32	10	45	27	52
Government	62	56	25	68	12	55	25	48
**Region**								
Eastern	67	60	21	57	12	55	34	65
Western	44	40	16	43	10	45	18	35

### Anthropometric Measurements

Body weight was measured to the nearest 0.1 kg using a digital weighing machine (Beurer Digital Glass Scale) while the participants were in school and wearing light uniforms. Height was measured to the nearest 0.1 cm using an electronic column scale (ADE Electronic) while the participants were in a full standing posture without shoes. The BMI was calculated as the ratio of the weight (kg) to height (m^2^) and assessed with the Saudi growth chart (shorturl.at/auwWZ)—a percentiles tool used to assess and monitor anthropometric measures in children (12).

### Scoring and Interpretation

Raven's Coloured Progressive Matrices test was used to assess IQ. It comprises three series—A, AB, and B—each including 12 items. Solving questions in Set A depends on the ability of the child to complete the continuing patterns. Solving questions in Set AB depends on the child's ability to perceive separate forms. Solving questions in Set B depends on the development of the child's ability in abstract thinking (13). Some scholars have argued for the testing of cultural generalizability. Nonetheless, a huge body of published research has confirmed test validity, reliability, and cross-cultural suitability (14).

The IQ score of the participants was transformed into a percentile according to the reference table for correction in the test manual. Percentiles lower than 5 signified intellectual deficiency; those between 6 and 25, below-average intellectual capacity; between 26 and 74, average intellectual capacity; between 75 and 94, above-average intellectual capacity; and higher than 95, gifted intellectual capacity.

The SI level was assessed using the Vineland Social Maturity Scale, which is considered a reliable and quick multidimensional instrument assessing social competency in eight social areas—self-help general, self-help eating, self-help dressing, self-direction, occupation, communication, locomotion, and socialisation (15). Despite its popularity, few researchers hold polarised views on the applicability of the Vineland Social Maturity Scale due to potential errors in administration and scoring (16).

The validity of Raven's Coloured Progressive Matrices was confirmed by internal consistency through the correlation between each subscale (A, AB, and B), and the total score ranged between 0.722 and 0.871, indicating the validity of the scale. The test also had a proper discriminant validity coefficient, calculated as the differences between the upper and lower values of the participants' IQ results by *t*-test (t = 11.259 and *P* = 0.001). Reliability was assessed by giving the scale to the same set of students on two occasions, 3 weeks apart. This test–retest method yielded a correlation of 0.939.

The Vineland Social Maturity assessment had a proper discriminant validity coefficient, as determined by calculating the differences between the upper and lower values of the SI level of the participants by *t*-test (*t* = 8.963 and *P* = 0.001). Reliability was assessed by giving the scale to the same set of students on two occasions, 3 weeks apart. This test–retest method yielded a correlation of 0.792.

Overall, 111 participants were included in the IQ analysis and BMI calculation comparison (*n* = 37, 22, and 52 for exclusively breastfed, exclusively bottle-fed, and mixed-fed, respectively). However, only 102 participants were included in the SI analysis (*n* = 37, 20, and 45 for exclusively breastfed, exclusively bottle-fed, and mixed-fed, respectively).

### Statistical Analysis

Data entry was conducted in Microsoft Excel, and the data were analysed using IBM SPSS Statistics, version 25 (17). Descriptive statistics and frequencies were used to describe the data, and a chi-square test was applied to the association analysis with a clustered bar plot for diagrammatic representation. Logistic regression analysis was performed as a risk analysis. Values of *P* < 0.05 were considered statistically significant.

## Results

### Description of Participants

Among the 111 participants, 37 (33.3%) were breastfed, 22 (19.8%) were bottle-fed, and 52 (46.8%) were mixed-fed. In this study, five students were underweight, 68 were normal weight, 17 were overweight, and 21 were obese. The IQ values were distributed as follows: 27 students had below-average scores, 50 had average scores, 32 had above-average scores, and two had gifted scores. Among the 102 participants in the SI analysis, one had a below-average score, five had average scores, four had above-average scores, 21 had superior scores, and 71 had very-superior scores.

Table 1 shows that the majority of participants were 8 years old (79%) and had normal BMIs (61%). Most participants reported moderate or high economic status, and 48% of the participants had mothers with a college level or higher education. Sixty-two (56%) of the participants were from governmental schools, and 67 (60%) were from the eastern region of the city.

### Comparison of IQ, SI, and BMI Among the Three Study Groups

**Figure 2:**
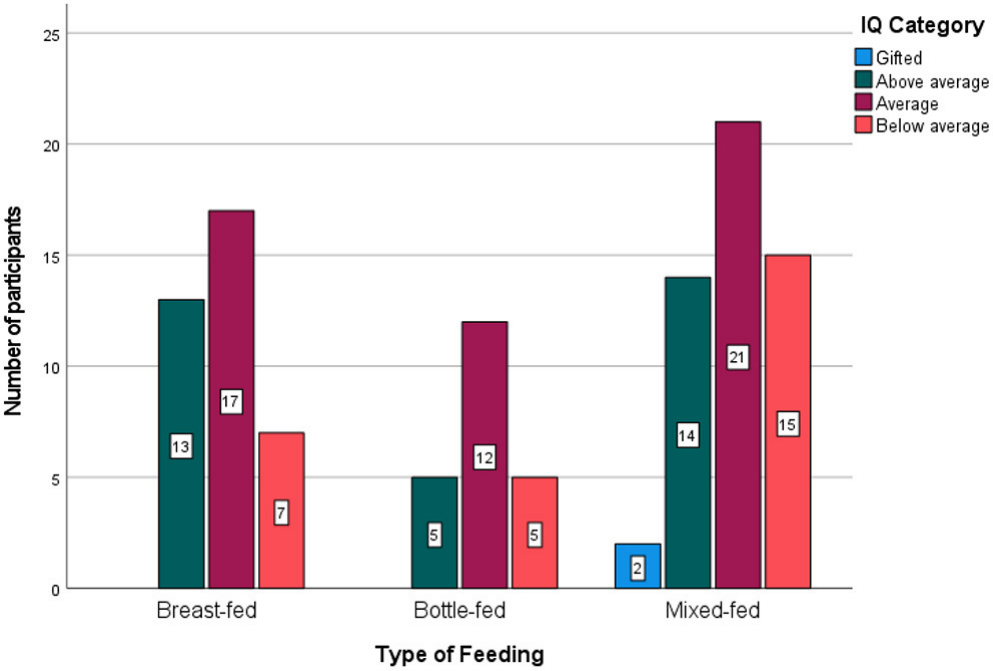
Intelligence quotient **(IQ)** levels among breastfed, bottle-fed, and mixed-fed groups.

The IQ levels among the three study groups were compared and are presented graphically in Figure 2. The percentage of participants with a below-average IQ was high in the mixed-fed group (29%), but an above-average IQ was more common in the breastfed group (35%). The statistical significance is presented in Table 2.

**Table 2:** Distribution of IQ, SI, and BMI results among participants.

**IQ levels**	**Breastfeeding ** ***N* (%)**	**Bottle feeding** ***N* (%)**	**Mixed feeding** ***N* (%)**	***P* value**
Gifted	0	0	2 (3.8)	0.579
Above average	13 (35.1)	5 (22.7)	14 (26.9)	
Average	17 (45.9)	12 (54.5)	21 (40.4)	
Below average	7 (18.9)	5 (22.7)	15 (28.8)	
**SI levels**				
Very superior	29 (78.4)	11 (55)	31 (68.9)	0.177
Superior	7 (18.9)	4 (20)	10 (22.2)	
Above average	1 (2.7)	1 (5)	2 (4.4)	
Average	0	3 (15)	2 (4.4)	
Below average	0	1 (5)	0	
**BMI Levels**				
Obese	5 (13.5)	6 (27.3)	10 (19.2)	0.435
Overweight	6 (16.2)	5 (22.7)	6 (11.5)	
Normal	25 (67.6)	9 (40.9)	34 (65.4)	
Underweight	1 (2.7)	2 (9.1)	2 (3.8)	

*BMI, body mass index; IQ, intelligence quotient; SI, social intelligence*.

**Figure 3:**
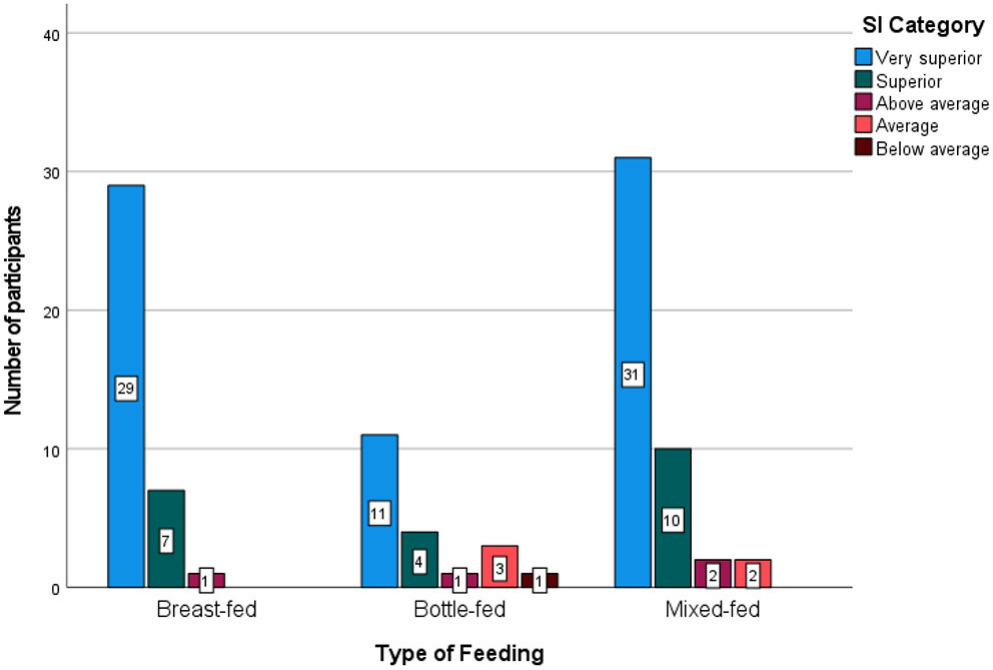
Social intelligence **(SI)** levels among the three feeding groups. Higher SI levels (>140) were seen more often in the breastfed group (78%) than in the other groups, and the lowest SI levels were noted in the bottle-fed group (in 5% of group participants). No low SI scores were noted in the other two groups.

Very-superior SI levels were noted more often in the breastfed group (78%) than in the bottle-fed (55%) and mixed-fed (69%) groups. In this study, no participant had average or below-average SI levels in the breastfed group, while in the bottle-fed group, 5% had below-average SI levels (Figure 3).

**Figure 4:**
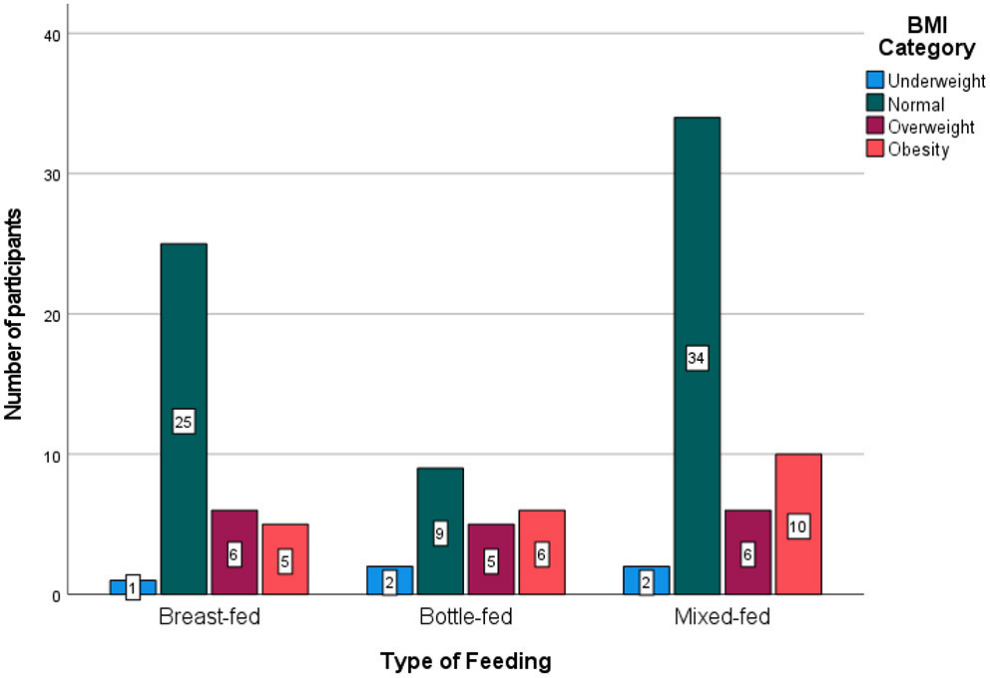
Body mass index **(BMI)** categories among the three feeding groups. The BMI measures were normal, more often in the breastfed group (68%) than in other groups (e.g., 41% in the bottle-fed group), and more obese BMI levels were seen in the bottle-fed group than in other groups (*P* = 0.435).

Body mass index measurements using the Saudi growth chart were defined and categorised into obese, overweight, normal, and underweight groups. Overall, 67.6% of the breastfed participants had a normal BMI, and 27.3% of the bottle-fed participants were obese (Figure 4).

Gifted or above-average IQ levels were noted more often in the breastfed group (35%) than in the bottle-fed (23%) or mixed-fed (31%) groups. Below-average IQ levels were seen more often in the mixed-fed group (29%) than in the breastfed group (19%).

Table 3 illustrates the IQ and SI results for the three study groups. An above-average IQ was more common in the breastfed group than in the bottle-fed group (35.1 vs. 22.7%), but this difference was not significant. Similarly, the SI result was high in the breastfed group more often than in the bottle-fed group (78.4 vs. 55.0%). Using univariate logistic regression analysis, it was found that breastfed children had a 2.24 higher chance of SI >140 as compared to bottle-fed children, which is not significant at the 5% level (*p*-value = 0.095).

**Table 3:** Association between feeding method and IQ or SI using logistic regression analysis.

**Feeding Method**	**IQ average or below**	**IQ above average**	**OR (95%CI)**	**p value**	**SI ≤140**	**SI > 140**	**OR (95%CI)**	***p*-value**
	***N* (%)**	***N* (%)**			***N* (%)**	***N* (%)**		
Bottle-fed	17 (77.3)	5 (22.7)	1.00		9 (45.0)	11 (55.0)	1.00	
Breastfed	24 (64.9)	13 (35.1)	2.24 (0.64–7.91)	0.209	8 (21.6)	29 (78.4)	2.81 (0.84–9.43)	0.095
Mixed-fed	36 (69.2)	16 (30.8)	1.65 (0.49–5.49)	0.418	14 (31.1)	31 (68.9)	1.67 (0.55–5.09)	0.370

*CI, confidence interval; IQ, intelligence quotient; OR, odds ratio; SI, social intelligence*.

## Discussion

Breastfeeding is the standard method of providing adequate nutrients for infants to achieve healthy growth and development. For mothers to breastfeed their newborns, they need constant support from all levels of society (https://apps.who.int/nutrition/topics/exclusive_breastfeeding/en/index.html).

### Intelligence Quotient

In 2006, a meta-analysis of prospective studies investigated the effect of breastfeeding on intelligence, which yielded 431 references and had a pooled sample size of 5,475 children. The research group concluded that breastfeeding had no significant effect on intelligence (18). On the other hand, a recent systematic review and meta-analysis were published in 2015, which included 17 studies and a total of 17,046 healthy breastfeeding infants, of whom 13,889 (81.5%) participated for 6.5 years. Breastfeeding was positively associated with IQ performance in children and adolescents. On an average, more breastfed participants had high IQ scores than non-breastfed participants (19). These findings agree with ours to some extent. However, because of the small sample size, we could not confirm the significant difference between the breastfed and bottle-fed groups (*P* = 0.579).

### Social Intelligence

According to Tasnim, mutual touch and the mother's gaze at her baby during breastfeeding may have positive effects on the child's psychological and social development. Breastfed children were found to be more cooperative and socially skilled (20). In the current study, breastfed participants reported higher SI scores than bottle-fed participants. Interestingly, bottle-feeding was the only category that reported SI levels of 80–90 (below average). Statistically, the result was significant at the 10% level (Table 3). Our findings mirror those published by Lind et al. (21). The study included 1,442 mother–child pairs and examined the association between breastfeeding duration and later psychosocial development in children aged 6 years. Breastfeeding children were divided into four groups: never breastfed; breastfed <6 months; breastfed ≥6 months, exclusively for <3 months; and breastfed ≥6 months, exclusively for ≥3 months. Mothers of the children reported strengths and difficulties in a questionnaire with domains similar to those of the Vineland Social Maturity Assessment. Results revealed that those who were breastfed ≥6 months and exclusively breastfed ≥3 months had lower odds of emotional difficulties than did those who were never breastfed (21).

### Body Mass Index

Childhood obesity is a serious public health concern. This study showed a significant difference in BMI between breastfed and bottle-fed children. In accordance with the present results, previous studies have demonstrated the beneficial effect of breastfeeding on a child's BMI in the first months of life. Owen et al. (22) conducted a systematic review of articles that investigated the effect of breastfeeding on mean BMI throughout life. Results showed that those who were breastfed had lower mean BMIs than those who were bottle-fed (22). Furthermore, a meta-analysis that included 25 studies with a total of 226,508 participants summarised the association between breastfeeding duration and childhood obesity. The results showed that a longer duration of the breastfeeding period was associated with a decreased risk of childhood obesity. Children breastfed for 7 months or longer were significantly less likely to be obese, whereas those breastfed for <3 months showed an approximate 10% decrease in the risk of childhood obesity (23).

After reviewing nationally available data on the prevalence of breastfeeding in Saudi Arabia, some researchers determined that breastfeeding was more dominant among rural mothers who breastfed for longer durations than among urban mothers and introduced complementary feeding methods later than urban mothers did. Research on breastfeeding in Saudi Arabia so far has been based on cross-sectional studies, and there is a need for cohort studies to measure breastfeeding and risk factors more accurately (3).

To our knowledge, this is the first study in a Saudi population to examine the effect of breastfeeding on IQ, SI, and BMI. However, our study also had some limitations. The sample size was relatively small. It also included only female participants due to limited accessibility. Although we have made efforts to control for potential confounders, there may still exist factors that could affect our results; for instance, the mixed-fed group definition was inconclusive. More specifically, the questions reflected a 6-month duration of breastfeeding, but some participants in the mixed-fed group were exclusively breastfed until 5 months old and continued breastfeeding thereafter with the addition of bottle feeding, while the others in the mixed-fed group were only breastfed for a few days and then were fully dependent on bottle feeding. This variance within the group ultimately led to an undefined proportion in the mixed-fed group, which could have undermined the results. To improve the reliability of the results, similar studies with larger sample sizes and with the inclusion of both genders are essential.

## Conclusion

Breastfeeding was positively correlated with both IQ and SI, yet the results were not statistically significant. However, there were significant differences in BMI between the breastfed and bottle-fed groups. This study provides basic observational data and can be modified for use on a national scale. The findings of our study shed light on the need for more research on the relationship between feeding modes and SI and IQ.

## Data Availability Statement

The original contributions presented in the study are included in the article/supplementary material, further inquiries can be directed to the corresponding author/s.

## Ethics Statement

This study was reviewed and approved by the Department of Clinical Nutrition and Institutional Review Board of Imam Abdulrahman Bin Faisal University and Ministry of Education. Written informed consent to participate in this study was provided by the participants' legal guardian/next of kin.

## Author Contributions

DA, FA, RA, and ZA conceived, planned the research, and carried out the simulations. TP contributed to the interpretation of the results. DA and FA took the lead in writing the manuscript. All authors provided critical commentary and helped in shaping the manuscript. All authors contributed to the article and approved the submitted version.

## Conflict of Interest

The authors declare that the research was conducted in the absence of any commercial or financial relationships that could be construed as a potential conflict of interest.

## Publisher's Note

All claims expressed in this article are solely those of the authors and do not necessarily represent those of their affiliated organizations, or those of the publisher, the editors and the reviewers. Any product that may be evaluated in this article, or claim that may be made by its manufacturer, is not guaranteed or endorsed by the publisher.
